# Physicochemical and Biological Properties of Menthol and Thymol-Based Natural Deep Eutectic Solvents

**DOI:** 10.3390/molecules30081713

**Published:** 2025-04-11

**Authors:** Martina Bagović Kolić, Martina Železnjak, Ksenija Markov, Višnja Gaurina Srček, Marina Cvjetko Bubalo, Kristina Radošević, Ivana Radojčić Redovniković

**Affiliations:** 1 Laboratory for Cell Technology, Application and Biotransformations, Department of Biochemical Engineering, Faculty of Food Technology and Biotechnology, University of Zagreb, 10000 Zagreb, Croatia; martina.bagovic@pbf.unizg.hr (M.B.K.); visnja.gaurina.srcek@pbf.unizg.hr (V.G.S.); marina.cvjetko.bubalo@pbf.unizg.hr (M.C.B.); ivana.radojcic.redovnikovic@pbf.unizg.hr (I.R.R.); 2 Laboratory for General Microbiology and Food Microbiology, Department of Biochemical Engineering, Faculty of Food Technology and Biotechnology, University of Zagreb, 10000 Zagreb, Croatia; ksenija.markov@pbf.unizg.hr

**Keywords:** hydrophobic DES, physiochemical properties, antioxidative capacity, antimicrobial activity, in vitro cytocompatibility, phytotoxicity

## Abstract

Seven hydrophobic deep eutectic solvents (hDESs) were characterised to evaluate their potential applicability in different industries and their environmental impact. Standard physicochemical properties were determined, yielding polarity and density values that were slightly higher for thymol-based hDESs than menthol-based ones, whereas for viscosity, the trend was opposite. Regarding biologically relevant activities, the antioxidative capacity and antimicrobial activity of hDESs were determined. Thymol-based hDESs are more potent as potential antioxidants, especially the one with coumarin as a hydrogen bond acceptor, which had the highest Oxygen Radical Absorbance Capacity (ORAC) value. Antimicrobial activity was assessed on four bacterial strains and one yeast strain. Calculated minimal inhibitory concentrations (MICs) showed that all hDESs possess this activity, and even the antimycotic effect against *C. albicans* was observed. Furthermore, to ensure the safety of these solvents for human use, in vitro cytocompatibility was determined. hDESs were tested on three human cell lines (HaCaT, CaCo-2, and HeLa), with no cytotoxic effect observed up to 1000 mg L^−1^. Finally, the environmental impact by the phytotoxicity test and in vitro antioxidative assay on wheat was determined for three selected hDESs, which were found to be slightly toxic, with different effects on plant defence mechanisms against induced antioxidative stress. Overall, the tested terpene-based hDESs demonstrate potential as alternative solvents for various industries, including food production, cosmetics, and pharmaceuticals, with thymol-based variants exhibiting a slight advantage in relation to the parameters evaluated in this study.

## 1. Introduction

A survey of the available literature since 2004, when Abbot et. al. [1] introduced deep eutectic solvents (DESs), until the present time points to a significant increase in the number of published manuscripts on that topic, both original scientific papers and reviews. Numerous studies explore DESs, focusing on their preparation, characterisation, applications, environmental impact, and sustainability [2,3,4,5,6]. Despite this extensive interest, the scientific community and process engineers remain deeply engaged in advancing green chemistry, achieving sustainable development goals, and fostering the industrial progress of DESs. The use of green solvents has become a prominent research focus, with DESs as promising alternatives to traditional organic solvents due to their simple preparation process, with 100% atom economy, low cost, biodegradability, and minimal toxicity [7]. However, the most often used DESs are highly miscible with water and hydrophilic organic solvents, significantly restricting their use in water-containing or lipophilic systems. To overcome that limitation and expand the range of DES applications, hydrophobic deep eutectic solvents (hDESs) have been introduced by van Osch et al. in 2015 [8]. They combined decanoic acid as a hydrogen bond donor (HBD) and quaternary ammonium salts as hydrogen bond acceptors (HBAs), offering hydrophobicity and excellent solvation capabilities for both organic and inorganic compounds. hDESs have been recognised as the fifth group of these 21st century green solvents, with a variety of uses, where their hydrophobicity can be exploited [9]. The reviews of van Osch et al. [10] as well as Cao and Su [11] highlight advancements in the properties and applications of hDESs across various fields, including extraction processes, biocatalysis, (enantio)separation, pharmaceuticals, energy utilisation, material modification, detection technologies for food safety and environmental protection, as well as liquid–liquid (micro)extractions, gas–liquid extractions, the formation of hydrogels, membrane formation, coating, photoluminescence, dye-sensitised solar cells, etc.

The first reported hDESs were successfully used for the extraction of volatile fatty acids from organic waste [8], showing that these innovative solvents perform better than the conventional extraction medium. In the second paper from a pioneer of hDESs, the ones based on terpenes were identified and characterised. Terpenes, like thymol and menthol, gained attention as a key constituent in hDESs due to their unique chemical properties and the fact that they are naturally occurring compounds. Thymol and menthol themselves are widely used in the food and pharmaceutical industries, according to specific regulations, which may vary in different countries. For example, clear guidelines are given in Belgium and France to ensure the safe usage of volatile or essential oils for food supplements [12]. Terpene interactions with drugs were extensively studied even before the emergence of hDESs, and explored for pharmaceutical applications, too. Some of them, like menthol and thymol, are listed as a pharmaceutical excipient, serving as flavouring agents and for topical applications. Therefore, their usage in medicinal products is already regulated by health authorities to ensure efficacy and safety [13], which can be advantageous in the context of utilising thymol- and menthol-based hDESs. Van Osch et al. [14] have screened many combinations based on terpenes, altogether five-hundred and seven, but only five of them finally satisfied the sustainability criteria and were used for the extraction of riboflavin from water. hDESs were also investigated for the removal of various biomolecules from water [15] as well as for the treatment of wastewater contaminated with various harmful compounds: bisphenol A, isopropanol, polycyclic aromatic hydrocarbons, and others [16,17]. Beyond the usage of hDESs to extract compounds from biological materials or aqueous media, they also hold promise as innovative solvents in the pharmaceutical industry, primarily for improving the solubility of active pharmaceutical ingredients (APIs). One effective approach is forming therapeutic deep eutectic solvents (THEDESs) by combining APIs with excipients like terpenes (e.g., menthol, thymol) or fatty acids (e.g., lauric acid), which liquefy APIs through hydrogen bonding [18,19,20,21]. Apart from liquefying APIs by forming THEDESs, another strategy for enhancing drug solubility in an array of studies is the design of hDESs as new drug vehicles [22]. Furthermore, it is noteworthy that DESs and hDESs inherently exhibit certain biological activities, highlighting their potential as diverse medicinal agents [23,24]. Among these, antimicrobial activity is the most extensively studied and of particular interest in both the food and pharmaceutical industries [25,26].

Whether the focus of the study was on the structure and physicochemical properties of DESs or their practical applications, the majority of investigated DESs are hydrophilic, with hDES-related publications accounting for less than 10% of DES studies [11]. This is primarily due to the limited availability of inexpensive and readily accessible hydrophobic salts or other components capable of forming hDESs. Based on their forming components, hDESs can be classified into two types. The first type are hDESs prepared from quaternary ammonium salts with long alkyl chains, while the second type comprises mixtures of two neutral hydrophobic components, such as carboxylic acids or alcohols with long alkyl chains. The presence of charged and polar parts in DESs lead to the formation of multiple hydrogen bonds, which consequently results in a greater depression of the melting point. In contrast, neutral hDESs exhibit a lower melting point depression and tend to form less viscous hDESs, which is a desirable characteristic from the applicative point [27]. The melting temperature of hDESs is generally below 25 °C and increases with the length of the alkyl chain or the content of fatty acid hydrogen bond donors. Employed hDESs are mostly characterised alongside their application. For example, an extraction of the environmental pollutant Cr (VI) from the aqueous phase into the hDES phase has been successfully performed [17]. In addition to its extraction potential, the electrochemical properties of a used hDES based on tetrabutylammonium chloride–decanoic acid (1:2 molar ratio) were also analysed, and it was shown that viscosity and electrical conductivity are strongly dependent on the water content.

From a chemical engineering perspective, the sustainability of hDESs has been evaluated using four key criteria introduced by Van Osch et al. [8]: viscosity, density, pH changes in the aqueous phase after mixing, and the extent of DES migration into the aqueous phase. It is well established that the physicochemical properties of DESs, including hDESs, can be tailored by carefully selecting their individual components based on molecular structure, chemical nature, molar ratio, and water content [8,28], allowing for their design to suit specific applications. However, the toxicity, biological activity, and biodegradability of DESs and hDESs are not necessarily predictable from their individual components alone. Studies have demonstrated that synergistic or additive effects can result in DESs that exhibit either enhanced or diminished potency compared to their constituents. Overall, the physicochemical and especially biological properties of hDESs remain relatively unexplored and warrant further investigation due to their higher efficiency and potentially broader range of applications compared to hydrophilic DESs. To address this gap, we prepared seven menthol- and thymol-based hDESs and characterised their physicochemical properties (polarity, density, and viscosity), as well as their antioxidative capacity, antimicrobial activity, in vitro cytocompatibility toward human cell lines, and phytotoxicity in wheat for three out of seven hDESs. This comprehensive analysis aims to provide deeper insight into their properties and promote their potential applications in fields such as food, cosmetics, pharmaceuticals, medicine, and environmental protection.

## 2. Results and Discussion

### 2.1. Physicochemical Properties of hDESs

DES properties must be characterised on a case-by-case basis. In our recent work on drug preparation and formulation, we utilise COSMO-RS to predict the solubility of active pharmaceutical ingredients in DES, based on the predicted logarithmic activity coefficient ln (γ) [20]. This approach identified menthol- and thymol-based hDESs as promising candidates, leading to their evaluation for physicochemical and biological properties. Physicochemical properties are crucial characteristics of DESs, as they directly influence their activity and end-use applications. In hydrophilic DESs, many properties—particularly viscosity and pH—can be modulated by adding water, which is advantageous when designing solvents for specific purposes [29]. Conversely, in the case of hDESs, the ability to alter properties is more limited, emphasising the importance of selecting an appropriate hDES and molar ratio based on their known properties. Table 1 presents the polarity, density, and viscosity of the tested hDESs.

**Table 1:** Physicochemical properties of hDESs at 25 °C.

hDES	Molar Ratio	Reference	Polarity [kcal mol^−1^]	Density [g cm^−3^]	Viscosity [mPa s]
Me:Cam	1:1	[10]	51.42	0.91	12.19
Me:C8	1:1	[10]	52.36	0.93	10.04
Me:C18:2	1:1	[30,31]	52.85	0.91	29.78
Me:Ty	3:2	[32]	52.08	0.93	27.47
Ty:Cou	3:2	[10]	52.17	1.07	17.92
Ty:C8	1:3	[3]	50.03	0.93	6.89
Ty:C10	1:1	[10]	50.87	0.94	9.72
H_2_O (literature data)	/		48.20	0.99	1.00

It is presumed that the polarity increases with stronger intermolecular attraction, making it a significant factor in the solubilisation efficacy of a solvent [11]. The polarity values presented here prove that all the hDESs are indeed non-polar, both by the colour of the solutions (Supplementary Materials Figure S1), shifting the bathochromic effect (red) to a pink colour rather than to a hypochromic effect (blue), and by the values, which are higher than 50 kcal mol^−1^. The highest values are obtained for menthol–octanoic acid (Me:C8), menthol–linoleic acid (Me:C18:2), menthol–thymol (Me:Ty), and thymol–coumarin (Ty:Cou), with a polarity close to that of MeOH (51.89 kcal mol^−1^). Menthol-based hDESs are less polar than thymol-based ones, except for Ty:Cou. The polarity of hDESs depends on both the properties of HBDs and HBAs, but the nature of the HBAs has a stronger impact on the overall polarity of hDESs. When it comes to the solvatochromic Nile red probe, calculated E_NR_ values for hDESs in the literature are between 50.34 and 52.6 kcal mol^−1^ [11,33,34]. Overall, as expected, hDESs have higher E_NR_ values than, for example, organic acid-based NADESs, which are the most polar (44.81 kcal mol^−1^), or even sugar and polyalcohol-based NADESs, which are the least polar among hydrophilic DESs [35].

The density is between 0.91 and 1.07 g cm^−3^, with the highest value for Ty:Cou, followed by thymol–decanoic acid (Ty:C10) > thymol–octanoic acid (Ty:C8) ≥ Me:Ty ≥ Me:C8 > Me:C18:2 ≥ menthol–camphor (Me:Cam). In general, the density is higher for thymol-based hDESs than menthol-based ones. These data are generally in correspondence with already reported data on hDESs [3,11,30]. Most hydrophilic DESs have a density higher than water (around 1.15 g mL^−1^), whereas the density range of hDESs is broader and mostly in the range between 0.88 g mL^−1^ and 0.97 g mL^−1^ [11].

The viscosity of DESs plays a crucial role in their potential applications and depends on factors such as temperature, hydrogen bond donor type, mixture composition, and shear rate [36,37]. The measurement of viscosity resulted in the highest values among the herein tested hDESs for Me:C18:2 and Me:Ty, 29.77 mPa s and 27.47 mPa s, respectively. The viscosity of other hDESs is lower, and the order is as follows: Ty:Cou > Me:Cam > Me:C8 > Ty:C10 > Ty:C8. The results on viscosity mostly follow the literature data, which report low viscosities of hDESs ranging from 2.31 to 1807 mPa S [11]. Our results on Me:Ty slightly differ from those reported in the work of Cao et al. [11]; these authors reported a value of 53.1 mPa s, but also for Ty:Cou; for which van Osch et al. [14] reported a value of 31.4 mPa s. These differences are probably due to different measuring temperatures, since temperature does influence viscosity [37]. Martins et al. [38] concluded that viscosity increases with the length of fatty acids and showed that thymol-based DESs have lower viscosity than menthol-based DESs, which agrees with our results. Viscosity is also influenced by the water content; for hDESs, the range reported in the literature is from 0.004 to 0.8 wt% [11]. That is generally lower than that of hydrophilic DESs, with neutral hDESs having an even lower water content than their ionic counterparts. The impact of water on the viscosity of hDESs is generally less pronounced than on hydrophilic DESs due to their lower water affinity; therefore, this was not assessed in this study. Furthermore, all tested hDESs meet the sustainability criteria introduced by van Osch et al. [14], i.e., their viscosity is below 100 mPa s, which enables their use as extraction solvents and all other processes where a lower viscosity is beneficial. Based on their rheological properties, DESs can be divided into two subclasses: Newtonian and non-Newtonian [39]. Typically, hDESs display non-Newtonian behaviour, which deviates from ideality due to the formation of hydrogen bonds [40]. Their viscosity is often shear rate-dependent, exhibiting either shear-thinning or shear-thickening behaviour. As shown in Supplementary Table S1, the calculated data indicate a decrease in viscosity with an increasing shear rate, which is a characteristic feature of non-Newtonian fluids [41].

Overall, hDESs tend to be far less viscous than the hydrophilic ones [42], thus making them easier to handle and more appropriate for industrial applications.

The investigated hDESs were not structurally characterised because the studied HBD/HBA molar ratios are already presented in the literature and referenced in Table 1 Also, when obtaining hDESs, the given combinations resulted in liquids, which aligns with the definition of DESs by Martinis et al. [43], Abranches and Coutinho [4] and Oyoun et al. [44]. These authors define DESs as eutectic-type systems that remain liquid at desired temperatures, where at least one component would otherwise be a solid and unsuitable as a solvent. According to them, DESs should not be limited to fixed HBD/HBA ratios but instead optimised for desired performance. Therefore, our study on prepared hDESs focused on the key physicochemical properties (polarity, density, and viscosity) relevant to their applications and their biological characterisation.

### 2.2. Antioxidative Capacity of hDESs

The antioxidative capacity of compounds, or in this case solvents, is an important factor which prevents the deleterious effects of Reactive Oxygen Species (ROS). Low to medium concentrations of ROS act as secondary messengers in intracellular signalling cascades and mediate the cell’s hormonal response; on the other hand, when homeostasis is disrupted, they are linked to many conditions like hypercholesterolemia, hypertension, diabetes, autoimmune disorders, rheumatoid arthritis, and many other diseases [45]. The antioxidative potential is already recognised as an additional value of some DESs. Such a property enables their inclusion in a variety of fields, such as cosmetics for antiaging [46,47], preservatives in food products, food supplements, and, when used as an extraction solvent, for plant components [29,48,49] and pharmaceuticals [25,50,51,52]. The results on the antioxidant capacity of hDESs are presented in Table 2. The smallest ORAC value was obtained for Me:Cam (16.22 µmol TE g^−1^), and the highest was for Ty:Cou at 4109.61 µmol TE g^−1^. All hDESs possess antioxidant potential and the order is as follows: Ty:Cou > Me:Ty > Ty:C10 > Ty:C8 > Me:C18:2 > Me:C8 > Me:Cam. Such an outcome is not surprising, since individual components of tested hDESs have antioxidative activity as well [53]. A notable difference is observed between the antioxidant capacity of menthol- and thymol-based hDESs, where ORAC values for thymol-based hDESs are 100 to 460 times higher. Such an outcome surely could be attributed to the forming compounds of hDESs, since the antioxidant capacity of menthol and thymol is already reported in the literature, and both compounds exhibit antioxidant properties, but their capacities vary [54]. For example, thymol has ORAC values ranging from 100 to 300 µmol TE g^−1^, depending on the concentration and matrix in which it is tested, whereas studies on menthol typically report lower ORAC values (from 20 to 60 µmol TE g^−1^) compared to thymol.

The literature data on the antioxidant activity of DESs are mostly about hydrophilic DESs, and the values are lower in comparison to hDESs. For example, ORAC values from 0.7 to 2.7 μmol TE g^−1^ are reported by Radošević et al. [55] for choline chloride-based DESs with alcohols, acids, amides, amines, or sugars as HDBs. The antioxidative capacity is most often related to the acidic components of DESs [56], or in general, it arises from the presence of components like polyols, organic acids, and amines, which are often natural antioxidants themselves.

The standard for the ORAC value is frequently debated, especially concerning the value that has a sufficient impact, and the ability of compounds to inhibit oxidative damage in biological systems. Reports from the USDA [57] claim that for dietary purposes, ORAC values from 3.000 to 5.000 µmol TE g^−1^ are considered to be sufficient for the organism to be protected from harmful oxidants. However, this classification should be accepted cautiously, since it was generously misused by the food and dietary supplements industry and is potentially outdated. If looking from that perspective, Me:Ty and Ty:Cou could be considered as a potential antioxidant for dietary purposes.

It can be concluded that thymol-based hDESs, particularly Ty:Cou, demonstrate a significant advantage for use in products intended to be marketed as antioxidants.

### 2.3. Antimicrobial Potential of hDESs

The antimicrobial activity of DESs is a unique property that has led them into the pharmaceutical world, where they have been utilised both for enhancing antibiotic properties in terms of solubility and permeability [50,58,59] and for a synergistic effect against bacteria. Elgharbawy et al. [25] have shown that the individual components of DESs and DESs themselves can act differently and that they have different effects on bacteria. It has also been reported that there might be a connection between the antibacterial activity and the lengthening of the fatty acid chain, where Watanaba et al. [60] have reported the antimicrobial activity of six saturated fatty acids and two unsaturated fatty acids against *S. aureus* and *S. epidermidis*. They place C8 (caprylic acid) and C10 (capric acid) in the non-selective antibacterial activity group, which exhibited antibacterial activity for *S. aureus* and *S. epidermidis*, and C18:1 (oleic acid), which could be considered similar to C18:2 (linoleic acid), in the group with preferential antimicrobial activity for *S. aureus*. They also reported on the minimal inhibitory concentration (MIC) of fatty acids, concluding that the MIC for saturated fatty acids decreases as the alkyl chain length increases. Elgharbawy et al. [25] noted the activity of hDESs (with MIC level 0.044 mg mL^−1^), where they outperformed the antibacterial effects of menthol, octanoic acid, and decanoic acid against all bacterial samples tested. Concerning the already proven activity of most individual components of hDESs, in the present work, we did not focus on individual components and possible synergistic effects on them, but only on the prepared seven hDESs (results in supplementary data, Tables S2 and S3). We observed that all of the hDESs had antimicrobial and antimycotic activity. The effect was similar to the effect of the used positive control. In comparison, menthol-based hDESs had slightly higher activity than thymol-based ones. Regarding the lengthening of the fatty chain, we have found they have similar effects when combined with thymol, but with a slight advantage to terpenoid camphor with menthol. Elgharbawy et al. [25] gave an interesting insight into the mechanism of how hDESs penetrate the membranes of bacteria. Gram-positive bacteria have thick, porous peptidoglycan layers that allow hDESs to penetrate and interact via hydrophobic interactions, leading to membrane disruption. In contrast, the complex outer membrane of Gram-negative bacteria, with lipopolysaccharides, blocks hDES penetration, thus making hDESs less effective against Gram-negative bacteria. Such an impact was not obvious from our results, since we obtained similar inhibition zones against both Gram-positive and Gram-negative bacteria. In the work of Rodrigues et al. [33], they tested the antimicrobial potential of an individual terpen-based NADES against *S. aureus* and *E. coli* as part of their study on the potential of NADESs to extract astaxanthin. They concluded that all NADESs were able to inhibit the growth of *S. aureus* and *E. coli*, with MICs ranging from 31.25 to 62.50 μL mL^−1^.

The MIC values in Table 3 represent a minimal concentration of hDESs that still have antibacterial activity. For *E. coli*, Me:Cam, Me:C8, Ty:C8, and Ty:C10 had the activity with the smallest MIC value, which was 0.006 mg mL^−1^. The same concentration (0.006 mg mL^−1^) of Me:MyA, Me:C8, Me:Ty, and Ty:Cou had an antimicrobial effect on *P. aeruginosa*, suggesting that based on MIC values, hDESs have a slightly stronger antimicrobial potential against Gram-negative bacteria than against Gram-positive bacteria, contrary to the presumed action based on the membrane structure [25]. The smallest inhibitory concentration on Gram-positive bacteria was detected to be 0.013 mg mL^−1^ for Me:Ty and Ty:C10 against *S. aureus*, as well as for Me:C8 and Ty:Cou against *L. monocytogenes*. The MIC value for the yeast strain *C. albicans*, for all tested hDESs, was 0.313 mg mL^−1^, apart from Me:C8 and Ty:Cou, with an MIC value at 0.625 mg mL^−1^. The highest antimycotic activity was observed with Me:Ty, with an inhibition zone of 12.33 mm, in comparison to the positive control (10.21 mm). In comparison to the findings of Radošević et al. [55], where no antimycotic activity was observed for hydrophilic NADESs, our results suggest that hDESs exhibit significantly greater potential in preventing mycotic diseases. To the best of our knowledge, the antimycotic activity of hDESs against *C. albicans* has not been previously reported in the available literature.

### 2.4. In Vitro Cytocompatibility of hDESs on Human Cell Lines

**Figure 1:**
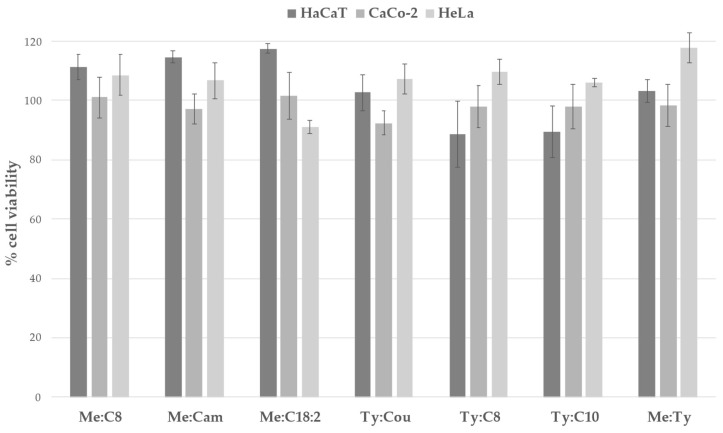
In vitro cytotoxicity expressed as % cell viability for 7 hDESs on three human cell lines in concentrations of 1000 mg L^−1^.

To evaluate in vitro cytocompatibility and safety for use in humans, we tested seven hDESs on three human cell lines: the keratinocyte-derived HaCaT cell line, the colon carcinoma-derived CaCo-2 cell line, and the cervical cancer-derived HeLa cell line. Currently, there are far more cytotoxicity assays performed with NADESs, where most of them have no or little cytotoxic effects on various cell lines [42,52,58,61]. The cytotoxic effects of hDESs have been less extensively studied, and the challenge of incorporating a hydrophobic component into hydrophilic cell culture media should not be overlooked. To improve the homogeneity of hDESs in aqueous cell growth media, 10% *v*/*v* ethanol (EtOH) must be added to the hDES before mixing with PBS. Notably, this concentration of EtOH has been shown to not affect cell viability [62]. The results presented in Figure 1 show no cytotoxic effect of hDESs in concentrations up to 1000 mg L^−1^ and lower (we have presented results only for the highest concentration), on either of the cell lines.

All of the tested hDESs had no antiproliferation effect on the cells, with the percentage of cell viability around 100%. Slightly lower viability was observed when treated with Ty:C8 and Ty:C10, where HaCaT cell viability was around 80%, and around 90% when the CaCo-2 cell line was treated with Ty:Cou. Given the abundance of the literature on the cytotoxicity of hydrophilic DESs, we can compare our results with those of Mitar et al. [42], who tested NADESs containing carboxylic acids on human HEK-293T, HeLa, and MCF-7 cells. They concluded that these NADESs were practically harmless, even at the highest concentration tested (2000 mg L^−1^), and considered them environmentally safe. Similar results were observed for several betain-based NADESs, which were investigated as alternative media for ocular applications, where most of NADES formulations did not significantly affect cell viability, with values generally above 80% [58]. However, there are also contrasting results regarding the cytotoxicity of hydrophilic DESs, such as those presented by Hayyan et al. [61], who investigated the cytotoxic potential of ammonium-based DESs. Their in vitro cytotoxicity study revealed that ammonium-based DESs exhibited relatively high cytotoxicity across various human cancer cell lines, with cytotoxicity levels being influenced by the composition, viscosity, and concentration of the DESs. This study also indicated that the cytotoxicity of DESs could be cell-line dependent, and the selectivity index varied among different DESs. Although data on the cytotoxicity of hDESs toward cell lines are relatively scarce, a recent study by Viñas-Ospino et al. [63] investigated the cytotoxicity of C8:Pro, Me:Eu, and C12:C8, as well as orange peel extracts obtained by those hDESs, on Caco-2 cells. The EC50 values for cytotoxicity, measured via the MTS assay, were approximately 21 μL mL^−1^. Additionally, they assessed the antiproliferative effects of these hDESs on HT29 cells, finding that the Me:Eu-based extract showed the greatest tumour cell selectivity. This is consistent with Cao and Su’s [11] findings, which highlighted that menthol-based hDESs have antiproliferative effects without harming normal cells. In the work of Rodrigues et al. [33] terpen-based NADESs were tested for antiproliferative effects on HT-29 cells and cytotoxic effects on the CaCo-2 cell line. The results showed that all NADES systems presented some toxicity in Caco-2 cells but were also able to inhibit the proliferation of HT-29 cells, with EC50 values of 0.89 and 3.67 mg mL^−1^ for CaCo-2 cells and 0.57 to 1.54 mg mL^−1^ for HT-2 cells. In contrast to our findings, we did not observe a significant antiproliferative effect of the tested hDESs up to a concentration of 1 mg mL^−1^, with higher concentrations remaining untested. Some studies [64] have indicated that hDESs may exhibit greater cytotoxicity than their individual components or physical mixtures. Nonetheless, based on in vitro tests on skin cell lines, including our own, the investigated hDESs can presumably come into contact with human skin. However, additional tests and precautions are needed, similar to those for their main components, menthol and thymol, which are limited by the concentration and administration route. Currently, there are no specific regulations for DES applications in the industry, and as noted in our recent paper, their use, for example, in the pharmaceutical field, is expected to fall under general frameworks by EMA and ICH [20]. Furthermore, the purification of the product obtained by DESs and recycling of DESs is also a potential option, since the recyclability of DESs is a significant factor in their potential application across various industries, but it needs to be further investigated in the future.

Accordingly, continued studies are necessary to assess the cytotoxicity of hDESs, as well as their biodegradability and environmental impact, particularly with regard to their effects on living organisms.

### 2.5. Phytotoxicity of hDESs

**Figure 2:**
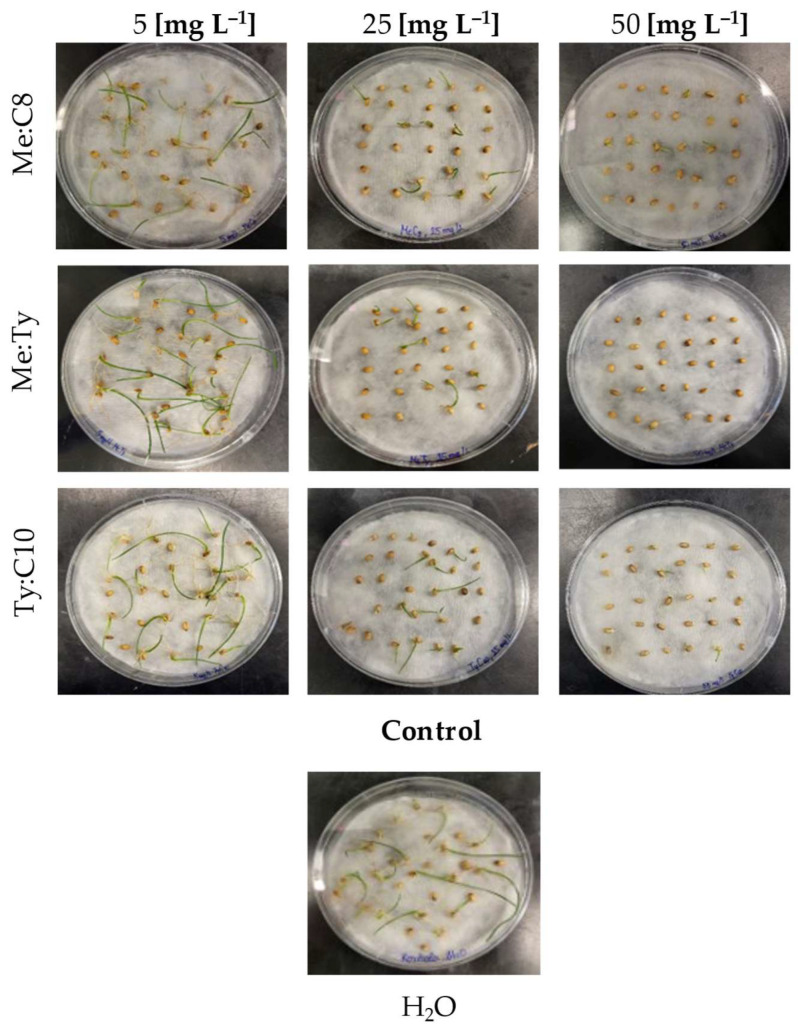
Shoots and roots growth on 7th day for 3 different concentrations of hDESs.

Phytotoxicity was assessed for three selected hDESs composed of menthol, thymol, and fatty acids, namely, Ty:C10 (1:1), Me:Ty (1:1), and Me:C8 (1:1). The plant material tested was wheat seeds (*Triticum aestivum* L.), a widely cultivated species with significant economic value. Germination in plants results from enzymatic reactions that activate anabolic and catabolic processes within the cell. When these processes are disrupted by the presence of xenobiotics, germination is inhibited. Furthermore, xenobiotic toxicity leads to slowed growth and reduced biomass accumulation, making shoot length a valuable indicator of phytotoxicity [65]. To better homogenise hDESs with demineralised water during the preparation of dilutions, EtOH is added to hDESs, the same as for the dilution in PBS for the cell viability assay. Ethanol itself is toxic to plants, as reported in previous research, according to which ethanol concentrations of 5% (*v*/*v*) and higher caused the significant inhibition of germination [66]. For that reason, only lower concentrations of hDESs with EtOH content that does not affect seed germination, i.e., less than 5%, were tested, and results after 7 days of treatment are presented in Table 4. Germination and shoot height inhibition were assessed as valuable growth parameters, and the corresponding half-maximal effective concentrations (EC50) were calculated (Table 5 and Figure 2).

In Table 4, the presented EC50 values are generally lower for shoot length, indicating that the toxic effects of hDESs are more pronounced on early growth than on seed germination itself, which is consistent with previous research by Radošević et al. [67] and Rodrigues et al. [68]. Radošević and colleagues investigated the toxic effects of DESs where the hydrogen bond acceptor is choline chloride (ChCl), which, unlike the hDESs tested in this study, is a hydrophilic solvent; so, much higher concentrations were tested (up to 20,000 mg L^−1^). Considering the classification proposed by Passino and Smith [69], the tested hDESs fall into the category of slightly toxic substances (10 to 100 mg L^−1^), whereas according to the same classification, Rodrigues [68] classifies choline chloride-based DESs as harmless (1000 mg L^−1^ or more).

In addition to growth parameters, the impact of hDESs on plant organisms was also assessed by examining antioxidant enzymes responsible for protecting plants from oxidative stress, which occurs under increased levels of environmental stressors. Antioxidant mechanisms are a fundamental physiological response of plants in contact with xenobiotics, resulting in modifications of the enzymatic activity of enzymes involved in these mechanisms. The antioxidant enzymes whose activities were determined include ascorbate peroxidase (APX), catalase (CAT), peroxidase (POD), and superoxide dismutase (SOD). These enzymes are responsible for maintaining homeostasis by reducing the concentration of ROS. Excessive ROS or failures in the antioxidant system can cause cell damage, making their removal by antioxidant enzymes essential [70]. The results are presented in Table 6.

SOD is considered to be the first line of defence in plants when exposed to high levels of O_2_^−^, eliminating it by conversion to H_2_O_2_ and O_2_. Consequently, high levels of H_2_O_2_ are also considered a stressor, and H_2_O_2_ triggers other scavenging enzymes for detoxifying, like CAT, POD, and APX [65].

The measured activity of the SOD enzyme is shown in Table 6. Compared to the control, a statistically significant decrease (*p* < 0.05) in activity was observed only in samples treated with Me:C8 at a concentration of 25 mg L^−1^, while other results do not differ statistically from the control. Radošević et al. [67] reported a positive correlation between SOD enzyme activity and DES Ch:OA concentration up to 1000 mg L^−1^, after which a decline in activity was noted. Similarly, Rodrigues et al. [68] observed a decrease in SOD enzyme activity with increasing concentrations of betaine-based DESs. Comparing these results with studies involving ionic liquids, which are precursors to DES in a green solvent design, a reduction in SOD activity with increasing concentrations, similar to the findings for Me:C8 in this study, was also reported by Chen et al. [71] while examining the effect of imidazolium-based ionic liquids on wheat. APX, the H_2_O_2_ scavenging enzyme, has a significant increase in activity in comparison to the control only when treated with 25 mg L^−1^ Ty:C10 and Me:Ty, while other concentrations of tested hDESs have similar effects as the control. When compared with the literature data, an increase in activity was also noted when plants were treated with Ch:OA (choline chloride–oxalic acid) and B:PG (betaine–polyethilen glycol) [67,68].

CAT and POD both catalyse the oxidation of H_2_O_2_ to H_2_O and O_2_. It has been reported [67,68] that when plants are treated with hydrophilic DESs, namely choline chloride and betaine-based DESs, a significant drop in CAT activity is observed. On the contrary, our results show that hDESs seem to have a different effect, since the CAT activity remains the same when treated with 5 mg mL^−1^ and is significantly higher when treated with 25 mg mL^−1^. This phenomenon suggests a different mechanism of response in the wheat defence system when exposed to different types of DESs.

As the name suggests, non-specific peroxidases (PODs) have low substrate specificity, and their activity is measured with the addition of guaiacol as an electron donor. A reciprocal increase in peroxidase activity was observed with the increase in the concentration of hDESs only in samples of plant material treated with Me:Ty, while in samples treated with other hDESs, the trend was the opposite. The measurement results are shown in Table 6. The highest activity of this enzyme was present in samples treated with Me:C8 at a concentration of 5 mg L^−1^, where a statistically significant increase was observed compared to the control (*p* < 0.05). In contrast to these results, Rodrigues et al. [68] observed a decrease in peroxidase activity in samples treated with betaine-based DESs, which diminished with increasing concentrations of certain DESs, while Radošević et al. [67] noticed an increase in non-specific peroxidase activity up to a concentration of 1000 mg L^−1^ Ch:OA, after which the activity decreased with further increases in DES concentration.

In summary, an increased activity of antioxidant enzymes has been observed as a result of oxidative stress caused by treatment with deep eutectic solvents. Increasing the concentration of hDESs in the treatment generally results in increased antioxidant enzyme activity, which is consistent with previous studies conducted with hydrophilic DESs [68]. Ascorbate peroxidase and catalase show increased activity in all samples treated with 25 mg L^−1^ hDES, while non-specific peroxidases show a decrease in activity in samples treated with Ty:C10 and Me:C8. The activity of superoxide dismutase does not change with increasing concentrations for Ty:10, while in samples treated with the other two hDESs, activity decreases. These results align with the premise [64] that the wheat defence mechanisms lead to different antioxidant and redox homeostasis responses to oxidative stress, depending on the examined hDESs, its concentration, and the type of radicals induced by the specific treatment.

## 3. Materials and Methods

### 3.1. Materials

Components for hDESs were purchased from Sigma-Aldrich (St. Louis, MO, USA), namely, (1*R*,2*S*,5*R*)-(-)-menthol (99%, CAS 2216-51-5); camphor (96%, CAS 76-22-2); octanoic acid for synthesis (CAS 124-07-2); thymol (≥98.5%, CAS 89-83-8); and linoleic acid (natural ≥ 95%, CAS 60-33-3). Decanoic acid (99%, CAS 334-48-5) and coumarin (99+% %, CAS 91-64-5) were purchased from Acros Organics (Newark, NJ, USA). Adherent human cancer cell line CaCo-2 (ATCC number: HTB-37™) and HeLa cervical adenocarcinoma (ATCC No. CCL-2™) were purchased from LGC Standards GmbH (Wesel, Germany), whereas adherent keratocyte cell line HaCaT (CLS number: 330493) was purchased from CLS Cell Lines Service GmbH (Eppelheim, Germany). Nile red was purchased from Sigma-Aldrich (CAS 7385-57-3). For the ORAC assay, 2,2′-Azobis(2-amidinopropane) dihydrochloride (AAPH) was purchased from Acros Organics (CAS 2997-92-4), New Jersey, USA; 6-hydroxy-2,5,7,8-tetramethylchroman-2-carboxylic acid (Trolox) was purchased from Sigma-Aldrich (CAS 53188-07-1), Steinheim, Germany; and Fluorescein was purchased from Sigma-Aldrich (CAS 2321-07-5), St. Louis, MO, USA.

### 3.2. Methods

#### 3.2.1. Preparation of hDESs

The seven hDESs listed in Table 7 were experimentally prepared by mixing the components in predicted molar ratios, stirred, and heated (50 °C), until a homogenous and stable liquid was formed.

#### 3.2.2. Polarity, Density, Viscosity

*Polarity* was assessed using Nile red as a solvatochromic probe and the method described by Ogihara et al. and Rodrigues et al. [68,72]. A Nile red stock solution was prepared (1.0 g L^−1^ in EtOH) and stored at 4 °C. The stock solution was diluted 100-fold in EtOH and added to the hDES, in a molar ratio of 1:3, after which the samples were placed in a 1 cm^3^ quartz cuvette. The absorption spectra of the dye (λ_max_) were obtained using a GENESYS 10S UV-Vis spectrophotometer, Thermo Fisher Scientific, Waltham, MA, USA. The molar transition energy (_ENR_) was calculated using the following formula:

E_NR_ (kcalmol^−1^) = 28591λ_max_^−1^ (1)

where λ_max_ is the maximum absorption wavelength in nm.

The *density* of hDESs was measured with a density meter DE40, Mettler Toledo, AG (Columbus, OH, USA), at 20 °C.

For the *viscosity* measurement, we used the rotational viscometer ViscQC 300, Anton Paar, GmbH (Graz, Austria). The driving torque is influenced by factors such as rotational speed, spindle geometry, and sample viscosity. After attaching the spindle to the rotational viscometer, the optimal speed for the measurement is set, and the viscosity is automatically calculated by the viscometer. These calculations account for the specific spindle used and the corresponding speed combination, eliminating the need for additional calculations. A standard spindle (L1) with a cylindrical shape was used at room temperature and the speed varied from 100 to 170 rpm. The sample volume was 2 mL, for which a CC12 12 mm cup was used. The shear rate was assessed by the following formula:

Shear rate (1 s^−1^) = SRC × speed [rpm] (2)

where SRC is the shear rate constant (for the measuring system CC12, SRC = 1.2908).

#### 3.2.3. Antioxidative Capacity Determined by ORAC Assay

Measurements were conducted with the spectrofluorimetric Oxygen Radical Absorbance Capacity (ORAC) assay using a Cary Eclipse Fluorescence Spectrofotometar (Varian, Mulgrave, Australia) and the protocol is described in detail by Mitar et al. [42]. In brief, fluorescein is used, a fluorophore that is susceptible to oxidation, which is induced by 2,2′-Azobis(2-amidinopropane) dihydrochloride (AAPH, acting as radical); when adding antioxidants, fluorescein is guarded, and the decay is delayed. A standard Trolox antioxidant (50 µmol L^−1^) and hDES samples are used to determine their potential antioxidant activity. All hDESs were prepared by adding 20 mg of hDES to a 10 mL mixture of 50% *v*/*v* acetone and water. Me:Cam, Me:C18:2, and Me:C8 did not require further dilution, Me:Ty was diluted 100-fold, as well as Ty:C8 and Ty:C10, while Ty:Cou required the highest dissolution of 1000-fold. The relative ORAC value is presented as μmol Trolox equivalent per gram of hDES (μmol TE g^−1^).

#### 3.2.4. Antimicrobial Activity Assay

Selected hDESs were tested against 4 bacterial strains to examine their potential for antimicrobial activity and *C. albicans* for antimycotic activity. Gram-positive bacterial strains, *S. aureus* (3048) and *L. monocytogenes* (3112), Gram-negative bacterial strains, *P. aeruginosa* (3024) and *E. coli* (3014), and a yeast strain, *C. albicans* (86), were obtained from the Microorganism Collection of the Laboratory for General Microbiology and Food Microbiology, University of Zagreb Faculty of Food Technology and Biotechnology (Zagreb, Croatia). Bacterial cultures were stored at −70 °C in a nutrient medium (Biolife, Milan, Italy). Strains were activated in corresponding broths and maintained at 4 °C. *C. albicans* was stored in yeast extract–peptone–glucose (YPG) medium composed of 2% glucose, 1% yeast extract, and 1% peptone (pH 5), with 30% (by volume) glycerol, respectively. The strains were activated in the same corresponding broths and maintained at 4 °C. The antimicrobial activity of hDESs was tested against selected microorganisms using the disk diffusion method. Microbial cultures were prepared using McFarland turbidity (Densimat, bioMérieux, Marcy-l’Étoile, France) with a 0.4 standard (10^7^–10^8^ CFU mL^−1^). Bacterial suspensions (0.1 mL) of each microorganism were spread on agar plates and allowed to dry under aseptic conditions. Sterile filter disks with a diameter of 6 mm (Macherey-Nagel GmbH, Düren, Germany) were immersed in the corresponding hDES in concentration ranges of 0.5—1 mg mL^−1^ for bacteria and 1 mg mL^−1^ for yeasts and placed on the surface of agar inoculated with the test microorganism. After the growth of microorganisms at 37 °C (bacteria) and 30 °C (yeast), the transparent zones around the disks were measured, meaning the growth was inhibited by hDESs. The positive control used in the same manner was 1 mg mL^−1^ of ciprofloxacin for bacteria and clotrimazole for yeast. The minimal inhibitory concentration (MIC) is a metric used to calculate the smallest concentration of a tested compound, i.e., hDES, that can have an antimicrobial effect and inhibit the growth of tested microorganisms. Bacteria and yeast cultures were prepared the same way as previously described in the work by Radošević et al. [55], and different decimal dilutions were prepared and tested.

#### 3.2.5. In Vitro Cytocompatibility

The in vitro cytocompatibility test was performed on three human cell lines; keratinocyte derived HaCaT cell line; colon carcinoma-derived CaCo-2 cell; cervical cancer-derived HeLa cell line, using a CellTiter96^®^ AQ_ueous_ One Solution Cell Proliferation assay based on MTS (3-(4,5-dimethylthiazol-2-yl)-5-(3-carboxymethoxyphenyl)-2-(4-sulfophenyl)-2*H*-tetra-zolium) (Promega, Madison, WA, USA), as stated by the manufacturer’s protocol. Cells were grown in BioLite Petri dishes by Thermo Fisher Scientific (Braunschweig, Germany) in DMEM supplemented with 10% (*v*/*v*) FBS (with exception CaCo-2 cell line, where 20% is added) and 1% (*v*/*v*) antibiotic/antimitotic solution, then trypsinzed and counted when needed using the trypan-blue method to maintain the good health of the cells and monolayer growth. All hDESs were prepared in a stock solution of 10 mg mL^−1^ hDES in PBS. Before adding the PBS, hDESs were mixed with 10% *v*/*v* EtOH, and the solution was stirred vigorously. For the MTS test, a volume of 100 µL was added to a 96-well plate with a starting concentration of cells 3 × 10^4^ cells mL^−1^. Added concentrations of hDESs ranged from 100 mg L^−1^ to 1000 mg L^−1^ and were left for over 72 h without changing the media. After the 3-day treatment, cell viability was tested by adding the MTS reagent for 3–4 h, and absorbance was measured at 492 nm on the microplate reader (Tecan, Männedorf, Switzerland). Cell viability percentage was expressed as the ratio between the absorbances of the treated and nontreated control cells. The tests were performed in triplicate and presented as a percentage of cell viability in comparison to nontreated cells.

#### 3.2.6. In Vitro Phytotoxicity on Wheat

The experimental system was set up as reported by Radošević et al. [67] and it consists of 30 wheat seeds per Petri dish, set up in 15 Petri dishes. The common wheat seeds (*Triticum aestivum* L.) were first sterilised by soaking them in a 1% sodium hypochlorite solution for 30 min, after which they were rinsed 3 times with sterile demineralised water and incubated in the dark for 24 h on filter paper. Petri dishes were coated with cotton in a uniform layer with a diameter of 15 cm, filter paper was placed, and 30 previously sterilised seeds were placed on top. Every dish was watered with 30 mL of the hDES (Ty:C10; Me:Ty; Me:C8) solution of a specified concentration (5, 25, and 50 mg L^−1^). The control group was set up with demineralised water. All experimental systems were incubated under controlled conditions: the temperature was 30 °C with a light/dark cycle (14/10 h). Every 24 h during the 7 days of cultivation, the length of shoots and roots were measured (results are presented for the 7th day), and at the end of the cultivation, plant material was collected. On the second and fifth days, the cotton was replaced, and 30 mL of DES/control solution was added to ensure constant contact of the seeds with the tested solvents and to maintain seed moisture.

#### 3.2.7. In Vitro Antioxidant Activity (APX, CAT, POD, SOD) in Wheat

After the growth and measurement of the shoot and root length, the shoots were homogenised using a mortar and pestle on ice. For extraction, 200 mg of plant material was weighed and homogenised with the addition of 2 mL of protein extraction buffer (pH = 7.0) and polyvinylpolypyrrolidone, a polymer used for protein stabilisation. The resulting extract was centrifuged at 10,000 rpm, decanted, and stored at –20 °C until analysis. This extract was used to determine the concentration of total soluble proteins using the Bradford method and to measure the activities of ascorbate peroxidase (APX), catalase (CAT), non-specific peroxidases (PODs), and superoxide dismutase (SOD). For all the tests, we used shoots that were treated with 5 and 25 mg L^−1^ hDES, since higher doses of hDESs did not yield enough biomaterial to set up the experiment.

APX measurement was performed according to the protocol by Ambriović Ristov [73] with minor modifications. In a quartz cuvette, 970 μL of ascorbate peroxidase activity buffer was pipetted, followed by 10 μL of Na-ascorbate and 10 μL of extract, after which 10 μL of hydrogen peroxide aliquot was added to initiate the reaction. The progress of the reaction was monitored spectrophotometrically at a wavelength of 290 nm every 15 s for 1 min. APX activity corresponds to the reduction in ascorbate with an extinction coefficient of 2.8 mM^−1^ cm^−1^ and is expressed as nmol per minute and mg of total protein (nmol min^−1^ mg^−1^).

For CAT, the start of the measurement was marked by the addition of 5 μL of the diluted sample to 995 μL of catalase activity buffer [73]. Activity was measured spectrophotometrically at a wavelength of 290 nm every 5 s for 1 min. The extinction coefficient is 40 mM^−1^ cm^−1^. Catalase activity is expressed as nmol per minute and mg of total protein (nmol min^−1^ mg^−1^).

To measure POD, 190 μL of non-specific peroxidase activity buffer was pipetted into a 96-well plate, and the reaction was initiated by adding 10 μL of extract. The increase in absorbance was monitored every 15 s for 5 min at a wavelength of 470 nm [73]. During the reaction, tetra guaiacol is formed, with a molar extinction coefficient of 26.6 mM^−1^ cm^−1^. Non-specific peroxidase activity is expressed as mmol per minute and mg of total protein (nmol min^−1^ mg^−1^).

For the SOD measurement, various dilutions of the extract were prepared by mixing specific volumes of the extract and protein extraction buffer (pH = 7.0) to a total volume of 33.3 μL when working in 96-well plates. To each well, 300 μL of superoxide dismutase activity buffer (pH = 7.8) and 0.67 μL of 2 μM riboflavin were added, and the enzyme reaction was initiated by turning on a 36 W light source. The reaction lasted for 10 min, after which it was stopped by covering the samples. Measurements were taken 5–10 min after the samples were covered using a spectrophotometer at a wavelength of 560 nm, after which an enzyme activity curve was created. SOD activity is expressed as the inhibition of substrate (NBT—nitroblue tetrazolium) reduction:

NBT (%) = [(A − B)/A] × 100 (3)

A—absorbance measured after the reaction without the enzyme (highest absorbance value); B—absorbance measured after the reaction with the enzyme (absorbance decrease).

The enzyme activity curve is constructed by plotting the NBT (%) reduction inhibition values on the *y*-axis, and the volumes of enzyme extract used for dilution preparation on the *x*-axis. The volume of enzyme extract required for the 50% inhibition of NBT in the reaction mixture is determined from the curve, and the specific SOD activity is calculated based on the total protein concentration, expressed in units of enzyme activity per milligram of total protein (U mg^−1^) [73].

#### 3.2.8. Statistical Analysis

Statistical analysis was conducted using SPSS Statistics software, version 2.0 (SPSS Inc., Chicago, IL, USA). Analysis of variance (ANOVA) followed by Tukey’s post hoc test was employed. Significance was determined at *p* < 0.05.

## 4. Conclusions

We evaluated seven menthol- and thymol-based hDESs, incorporating different fatty acids and terpenoids as HBAs, in terms of their physicochemical properties, in vitro cytocompatibility on human cell lines, and phytotoxicity on wheat, for three out of seven hDESs. Additionally, their antimicrobial activity was assessed against four bacterial strains and one yeast strain. Our study revealed promising bioactive properties and highlighted intriguing mechanisms of the wheat defence system when exposed to hDESs.

The antioxidant activity, measured through ORAC assays, showed that thymol-based hDESs, particularly Ty:Cou, have an exceptionally high antioxidative potential, making them viable candidates for use in dietary and cosmetic applications. As for the in vitro cytocompatibility test on three cell lines, namely HaCaT, HeLa, and CaCo-2, we observed no cytotoxic effect when treated with hDESs, making them safe for use in humans. The antimicrobial tests against various bacterial and yeast strains highlighted that hDESs possess inhibitory antibacterial activity, making them interesting for use against antibiotic resistance and possible use in environmental protection, and adding value to existing antibiotics. From an environmental perspective, and considering possible leaching properties upon disposal in nature, another advantage of the tested hDESs is that they do not inhibit superoxide dismutase activity in the plant defence system against oxidative stress. In vitro phytotoxicity has indicated that hDESs should be categorised as slightly toxic substances, with a more pronounced effect on early growth than on seed germination itself.

These findings underscore the potential of hDESs in fields such as medicine, food, environmental protection, and cosmetics. However, further research is required to fully understand their mechanisms of action, optimise their formulations, and assess their long-term safety and environmental impact to achieve their responsible and sustainable application.

## Figures and Tables

**Table 2:** ORAC values of tested hDESs.

hDES	Molar Ratio	ORAC Value [µmol TE g^−1^]
Me:Cam	1:1	16.22 ± 1.76
Me:C8	1:1	12.75 ± 0.16
Me:C18:2	1:1	43.32 ± 2.61
Me:Ty	3:2	2279.97 ± 103.09
Ty:Cou	3:2	4109.61 ± 80.0
Ty:C8	1:3	1604.15 ± 50.35
Ty:C10	1:1	1152.75 ± 15.79

**Table 3:** MIC values in mg mL^−1^ on different bacterial and yeast strains.

Bacterial Strain	Hdes						
	Me:Cam (1:1)	Me:C8 (1:1)	Me:C18:2 (1:1)	Me:Ty (3:2)	Ty:Cou (3:2)	Ty:C8 (1:3)	Ty:C10 (1:1)
*E. coli*	0.006	0.006	0.050	0.050	0.025	0.006	0.006
*P. aeruginosa*	0.025	0.006	0.013	0.006	0.006	0.063	0.031
*S. aureus*	0.031	0.031	0.063	0.013	0.063	0.025	0.013
*L. monocytogenes*	0.100	0.013	0.063	0.031	0.013	0.250	0.125
*C. albicans*	0.313	0.625	0.313	0.313	0.625	0.313	0.313

**Table 4:** Average shoot length (mm) and % germination after 7 days of cultivation in hDESs.

hDES	mg L^−1^	Average Shoot Length (mm)	% Germination (vs. Control)
Ty:C10 (1:1)	5	34.1 ± 4.7	105.9
	25	18.4 ± 3.7	73.8
	50	5.9 ± 0.7	0.8
Me:Ty (1:1)	5	38.3 ± 2.0	131.6
	25	17.9 ± 3.4	47.4
	50	5.0 ± 0.8	10.6
Me:C8 (1:1)	5	24.9 ± 3.3	105.6
	25	15.8 ± 2.4	73.8
	50	6.6 ± 1.1	52.6
Control (H_2_O)	/	31.9 ± 5.9	100

**Table 5:** EC50 [mg L^−1^] values for shoots and roots.

hDES	Shoots	Roots
Ty:C10 (1:1)	77.50	28.79
Me:Ty (1:1)	24.13	27.52
Me:C8 (1:1)	50.18	24.62

**Table 6:** Results of antioxidant enzyme activity at the concentration of 5 mg L^−1^ and 25 mg L^−1^ of hDESs.

hDES	POD [nmol min^−1^ mg^−1^]		SOD [U mg^−1^]		APX [nmol min^−1^ mg^−1^]		CAT [nmol min^−1^ mg^−1^]	
Conc. [mg mL^−1^]	5	25	5	25	5	25	5	25
Ty:C10 (1:1)	188.10 ± 6.205	171.79 ± 0.85	28.525 ± 1.797	29.42 ± 2.54	3.945 ± 0.318	6.85 ± 0.073	0.0445 ± 0.009	0.087 ± 0.003
Me:Ty (1:1)	141.809 ± 2.159	182.89 ± 2.31	25.805 ± 0.379	24.83 ± 0.52	4.698 ± 0.095	5.89 ± 0.115	0.0585 ± 0.009	0.08 ± 0.006
Me:C8 (1:1)	196.8 ± 2.512	156.51 ± 1.98	24.571 ± 1.149	21.08 ± 1.25	3.268 ± 0.178	5.52 ± 0.009	0.051 ± 0.009	0.07 ± 0.002
Control (H_2_O)	167.71 ± 5.9		28.38 ± 0.49		4.24 ± 0.277		0.05 ± 0.008	

**Table 7:** List of prepared hDESs.

Full Name hDES	Abbreviation	Molar Ratio
Menthol–Camphor	Me:Cam	1:1
Menthol–Octanoic acid	Me:C8	1:1
Menthol–Linoleic acid	Me:C18:2	1:1
Menthol–Thymol	Me:Ty	3:2
Thymol–Coumarin	Ty:Cou	3:2
Thymol–Octanoic acid	Ty:C8	1:3
Thymol–Decanoic acid	Ty:C10	1:1

## Data Availability

Additional data are available on request.

## Supplementary Materials

The following supporting information can be downloaded at: https://www.mdpi.com/article/10.3390/molecules30081713/s1, Figure S1. Polarity of hDESs, Table S1: viscosity, rpm, and shear rates of hDESs, Table S2: antimicrobial activity of hDESs, Table S3: antimicotic activity of hDESs.
