# Supplementary material for: Physicochemical and Biological Properties of Menthol and Thymol-Based Natural Deep Eutectic Solvents
Source: Molecules. 2025 Apr 11;30(8):1713. doi: 10.3390/molecules30081713 (PMC12029795; doi:10.3390/molecules30081713)
Supplement: Supplementary file 1 [file molecules-30-01713-s001.zip › molecules-3505055-supplementary.pdf]

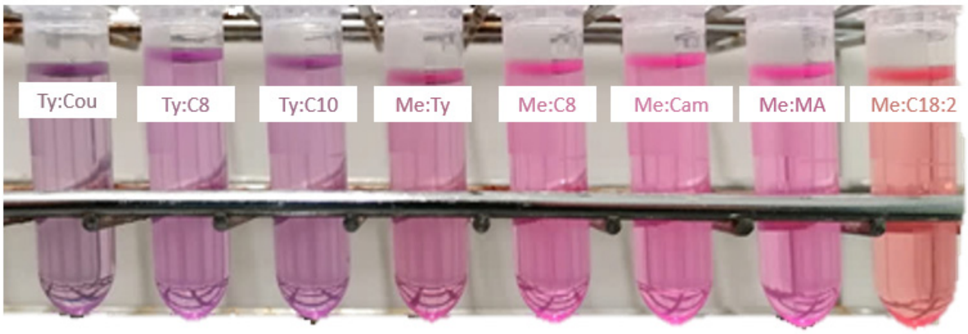

Figure S1. Polarity of hDES

Table S1. Viscosity, rpm and shear rate of hDES

hDES	Viscosity [mPa s]	Speed [rpm]	Shear rate [1 s^{-1}]
Me:Cam	12.19	100	129.08
Me:Ty	27.47	100	129.08
Me:C8	10.04	120	154.896
Me:C182	29.78	100	129.08
Ty:Cou	17.92	100	129.08
Ty:C8	6.89	170	219.44
Ty:C10	9.72	120	154.90

Table S2. Antimicrobial activity of hDES (inhibition zones in mm)

hDES (1 mg mL^{-1})	*S. aureus*	*L. monocytogenes*	*P. aeruginosa*	*E. coli*
Me:Ty	10 ± 0	10 ± 1.41	9 ± 0	9.50 ± 0.71
Me:Cam	9.50 ± 2.12	8.50 ± 2.12	8 ± 0	7.50 ± 0.71
Me: C18:2	8 ± 0	8 ± 0	7 ± 0	9 ± 0
Me:C8	7 ± 0	9 ± 0	8.50 ± 0.71	7 ± 0
Me:MA	9.50 ± 2.12	10.50 ± 0.71	8 ± 0	7.50 ± 0.71
Ty:Cou	8.50 ± 0.71	8 ± 0	8.50 ± 0.71	8.50 ± 0.71
Ty:C10	8.00 ± 1.41	8.50 ± 0.71	8 ± 0	8.50 ± 0.71
Ty:C8	8.50 ± 0.71	8 ± 0	7.50 ± 0.71	7.50 ± 0.71
Positive control (1 mg mL^{-1} antibiotic)	9.28 ± 1.76	12.33 ± 1.1	8.233 ± 0.38	8.833 ± 0.38

Table S3. Antimicotic activity of hDES on *Candida albicans* (inhibition zones in mm)

hDES (5 mg mL^{-1})	*C. albicans*
Me:Ty	12.33 ± 2.89
Me:Cam	8 ± 1
Me: C18:2	8 ± 0
Me:C8	9.33 ± 1.15
Me:MA	7.67 ± 1.15
Ty:Cou	10.33 ± 3.51
Ty:C10	11.67 ±2.52
Ty:C8	11.66 ±3.21
Positive control (5 mg mL^{-1} antimycotic)	10.21 ± 0.55
